# Constitutive basal and stimulated human small bowel contractility is enhanced in obesity

**DOI:** 10.1186/1750-1164-3-4

**Published:** 2009-04-20

**Authors:** Tom K Gallagher, Alan W Baird, Desmond C Winter

**Affiliations:** 1 Institute for Clinical Outcomes, Research and Education (ICORE), St Vincent's University Hospital, Elm Park, Dublin 4, Republic of Ireland; 2 Department of Physiology, College of Life Sciences, University College Dublin, Dublin, Republic of Ireland; 3 Department of Surgery, St Vincent's University Hospital, Elm Park, Dublin 4, Republic of Ireland

## Abstract

Small bowel contractility may be more prominent in obese subjects, such that there is enhanced nutrient absorption and hunger stimulation. However, there is little evidence to support this. This study examined in vitro small bowel contractility in obese patients versus non-obese patients.

Samples of histologically normal small bowel were obtained at laparoscopic Roux-en-Y gastric bypass from obese patients. Control specimens were taken from non-obese patients undergoing small bowel resection for benign disease or formation of an ileal pouch-anal anastamosis. Samples were transported in a pre-oxygenated Krebs solution. Microdissected circular smooth muscle strips were suspended under 1 g of tension in organ baths containing Krebs solution oxygenated with 95% O_2_/5% CO_2 _at 37°C. Contractile activity was recorded using isometric transducers at baseline and in response to receptor-mediated contractility using prostaglandin F_2a_, a nitric oxide donor and substance P under both equivocal and non-adreneregic, non-cholinergic conditions (guanethidine and atropine).

Following equilibration, the initial response to the cholinergic agonist carbachol (0.1 mmol/L) was significantly increased in the obese group (n = 63) versus the lean group (n = 61) with a mean maximum response: weight ratio of 4.58 ± 0.89 vs 3.53 ± 0.74; (p = 0.032). Following washout and re-calibration, cumulative application of substance P and prostaglandin F2a produced concentration-dependent contractions of human small bowel smooth muscle strips. Contractile responses of obese small bowel under equivocal conditions were significantly increased compared with non-obese small bowel (p < 0.05 for all agonists). However, no significant differences were shown between the groups when the experiments were performed under NANC conditions. There were no significant differences found between the groups when challenged with nitric oxide, under either equivocal or NANC conditions.

Stimulated human small bowel contractility is increased in obese patients suggesting faster enteric emptying and more rapid intestinal transit. This may translate into enhanced appetite and reduced satiety.

## Introduction

The development of obesity results from a person's inadequate energy expenditure and/or excessive caloric intake. In relation to caloric intake, the gastrointestinal (GI) system plays a vital role in the controlling nutrient ingestion, digestion, and absorption. These functions depend on an intact, coordinated GI motility, which not only regulates the rates at which nutrients are processed but also participates in the control of appetite and satiety. Alterations in GI motility have been observed in obese patients [1,2], and these alterations could be contributing factors to the development and maintenance of obesity and changed eating behaviors.

There are conflicting reports regarding the role of the small intestine in obesity. Two major areas of relevance to the whole debate are small intestinal motility and absorption of nutrients. Intestinal transit plays a crucial role in the absorption of nutrients. Variations in intestinal motility control the transit and absorption of the ingested nutrients through negative feedback [3,4] and hormonal methods [5], thus affecting satiety. On the one hand, if obese patients demonstrate increased intestinal motility and transit, does this indicate that they have enhanced absorptive capacity? However, if they have a decreased transit time, what factor is present that blocks the negative feedback on satiety in these patients? It has been speculated that the intestinal absorption is more rapid and efficient in obesity, irrespective of the intestinal transit rate, but the supporting evidence is very limited.

There are very few reports in the literature relating to investigation of small intestinal motility in the obese. It has been suggested that the contractile activity of small intestine is more prominent in the fasting period in obese subject [6]. Pieramico et al suggested motility disturbances in the fasting period, including diminished Phase I, increased Phase II, and a more distal and less frequent occurrence of Phase III activity of the migrating motor complex in association with decreased plasma motilin concentrations. The significance of these changes is not fully understood. They may also suggest that the contractile activity of small intestine is more prominent in the fasting period in obese subjects. This may be associated with more efficient absorption of nutrients within the small intestine or the precipitation of hunger.

The aim of this study was to demonstrate the differences, if any, between in vitro contractility, stimulated by common agonists, of small bowel from morbidly obese patients and that of patients with a normal BMI.

## Methods

### Patients and tissue preparation

The University Hospital's ethics committee and institutional review board approved this prospective study and the use of human tissue.

Specimens of histologically normal human small bowel smooth muscle were obtained at the time of laparoscopic Roux-en-Y gastric bypass from eight obese patients (5 female, 3 male) with a mean body mass index (BMI) of 48.2 kg/m^2 ^(range 44.2 kg/m^2 ^– 60.3 kg/m^2^), and a mean age of 44.25 years (range 29.1 – 60.8 years). Eight control small bowel specimens were taken from eight non-obese patients (4 female, 4 male) undergoing either small bowel resection for benign disease (five patients) or formation of an ileal pouch-anal anastomosis (three patients). The mean BMI of the controls was 24.1 kg/m^2 ^(range 20.8 kg/m^2 ^– 25.4 kg/m^2^), with a mean age of 38.75 years (range 20.3 – 52.6 years). In the case of specimens resected from patients with Crohn's disease, the specimen was obtained from an area proximal to the affected site and was uninvolved histologically. None of the patients had diabetes or a known motility disorder.

The specimens were transported to the laboratory in an oxygenated cold Krebs solution (4°C) and all experiments were started within one hour of receiving the specimen. The muscle was stripped of the underlying mucosa and submucosa, and from each specimen eight strips of circular smooth muscle approximately 0.5 cm wide × 1.0 cm long were mounted in 10-ml organ baths containing Krebs solution of the following composition (mmol/L): NaCl, 118; KCl, 4.7; MgSO_4_, 1.2; KH_2_PO_4_, 1.2; glucose, 11.1; NaHCO_3_, 24.9, and CaCl_2_, 2.5, maintained at 37°C and gased with 95% O_2 _and 5% CO_2_. A resting preload of 1 g was applied to each muscle strip, which was then allowed to equilibrate for one hour. During this time the Krebs solution was changed every 20 min. Mechanical activity was recorded using isometric transducers (World Precision Instruments, Stevenage, Herts, UK). Tension was continuously monitored and recorded using a MacLab data acquisition system (AD Instruments, Hastings, UK).

### Experimental protocol

After an equilibration period of one hour, the tissues were exposed to carbachol (0.1 mmol/L) to determine the maximal contractile capacity. This carbachol was then washed out and the preparations left to re-equilibrate for approximately 30 minutes. After this equilibration period, baseline contractility of the specimens was observed and recorded for a further 30 minutes.

Receptor-mediated contractions to prostaglandin F_2a _(0.01 nmol/L – 1 μmol/L), a nitric oxide donor in the form of Deta-nonoate (NOC18, 0.01 nmol/L – 1 μmol/L) and substance P (0.01 nmol/L – 1 μmol/L) were then studied. In a second set of experiments, inhibitory non-adrenergic non-cholinergic (NANC) neurotransmission was studied in separate samples of the same specimens. The NANC conditions were established by adding guanethidine (3 μmol/L) and atropine (1 μmol/L) to the organ bath. Receptor-mediated contractions to the above agonists were then studied separately under these conditions.

All strips were challenged with carbachol (0.1 mmol/L) towards the end of the experiment to ensure viability and subsequently the response to 1 μmol/L atropine was determined for each strip at the end and used as reference for calculating the responses to the test substances. At least one preparation for each specimen was used as control (tissue incubated with the test substance vehicle only). The tissue was destroyed following experimentation under the terms of the local guidelines and procedures.

### Chemicals

Carbachol, prostaglandin F_2a_, and substance P were all purchased from Sigma-Aldrich (Dublin, Ireland). Deta-nonoate (NOC18) was kindly donated by Professor Cormac Taylor (Conway Institute of Biomolecular and Biomedical Research, University College Dublin, Ireland).

### Statistical analysis

Results are expressed as percentage of the maximum carbachol response. Data are presented as mean ± SEM. Comparisons between contractile responses were made using Student's unpaired two-tailed t test or analysis of variance, when applicable. Differences between groups were taken to be significant if p < 0.05. The agonist/antagonist concentration producing 50% maximal effect (EC_50 _or IC_50_), with adjustment by non-linear regression was calculated using GraphPad Prism version 5.00 for Windows, GraphPad Software, San Diego, California, USA.

## Results

**Figure 1:**
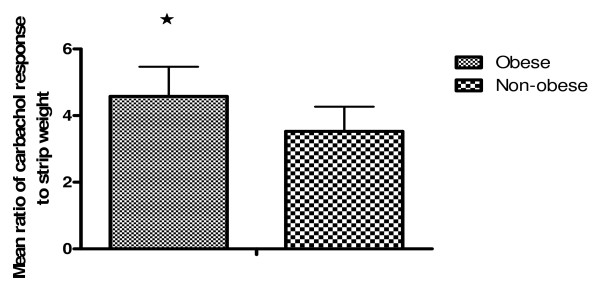
**Representation of the significant differences in contractility between small bowel smooth muscle strips from obese and non-obese patients**. The values given represent a mean value of the ratio of maximum carbachol response for each strip to its weight, and the difference is significant. 4.39 ± 0.82 (n = 63) in the small bowel muscle strips from obese patients versus 3.87 ± 0.84 (n = 61) in the strips from control patients. (p = 0.029).

A maximally effective concentration of carbachol (0.1 mmol/L), measured as a ratio to the weight of each strip produced a ratio of 4.39 ± 0.82 (n = 63) in the small bowel muscle strips from obese patients. In comparison, the mean ratio for the controls was 3.87 ± 0.84 (n = 61) and the difference was shown to be statistically significant (p = 0.029) (Figure 1).

For both sets of experiments, the response to carbachol was initially used as an indicator of the capacity of individual tissues to contract. Responses to the further agonists were then expressed as a percentage of the maximum carbachol response for each strip, and a mean percentage response at each concentration was than recoded.

**Figure 2:**
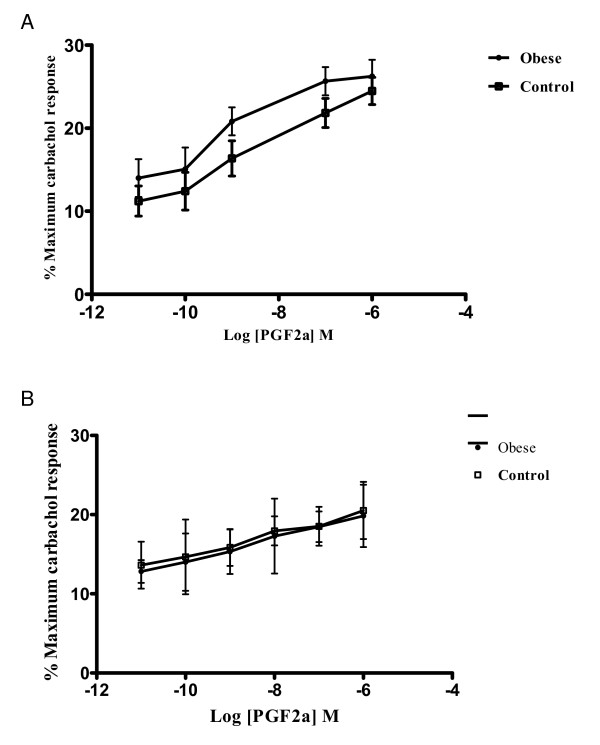
**A. Logarithm concentration-response curves of prostaglandin F2a as measured from strips from obese and control patients**. Results are the mean (SD) of 6–8 preparations from different patients, with the response expressed as a percentage of the maximal effect of atropine (1 umol/L). The differences demonstrated are statistically significant. B. Logarithm concentration-response curves of prostaglandin F2a as measured from strips from obese and control patients under NANC conditions. Results are the mean (SD) of 6–8 preparations from different patients, with the response expressed as a percentage of the maximal effect of atropine (1 umol/L). There is no statistically significant difference between the groups.

Prostaglandin F2a (Figure 2a) produced a concentration-dependant increase in contractility as expected. In obese tissues and under equivocal conditions, prostaglandin F2a was twice as potent in effecting a contractile response as it was in the control strips, with an EC_50 _(95% confidence limits) of 0.74 nmol/L (0.41 – 1.33) and 1.380 nmol/L (0.76 – 2.56) respectively (p < 0.01).

Under NANC conditions (Figure 2b), there was no significant difference between the obese strips and the controls when PGF2a was added in cumulative concentrations with an EC_50 _of 3.09 nmol/L (0.31 – 29.91) and 3.05 nmol/L (0.20 – 46.13) respectively.

**Figure 3:**
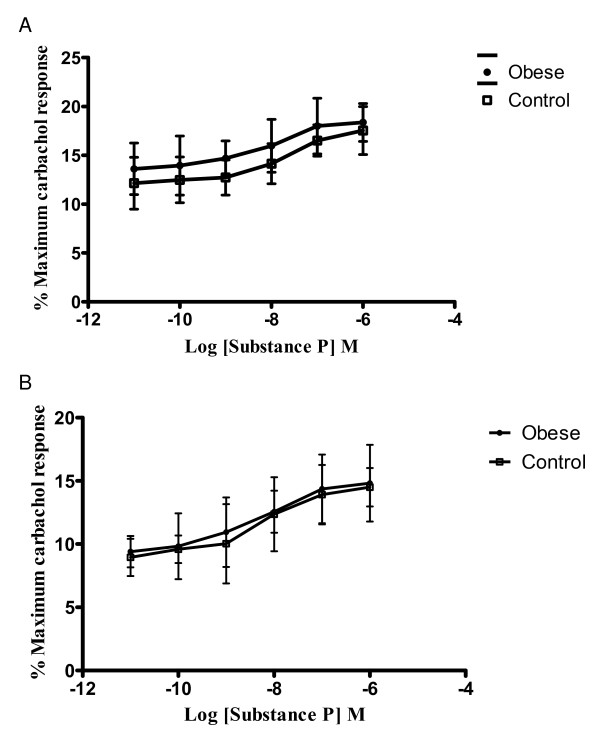
**A. Logarithm concentration-response curves of substance P in strips from obese and control patients**. Results shown are the mean of 6–8 strips from differenct patients, and the response is expressed as a percentage of the maximum response to carbachol (0.1 mmol/L). The differnces demonstrated are statistically significant. B – Cumulative concentrations of substance P have similar potency in obese and control tissues under NANC conditions. Results shown are a mean of 6–8 patients in each group, expressed as a percentage of the maximum response to carbachol (0.1 mmol/L). There are no statistically significant differences demonstrated under NANC conditions.

Substance P (Figure 3a) also produced a concentration-dependant increase in contractility as expected. The addition of substance P produced a contractile response in strips from obese patients that was again roughly twice that of the response in the controls with an EC_50 _of 10.42 nmol/L (3.81 – 28.47) and 20.09 nmol/L (10.90 – 37.00) (p < 0.01).

Under NANC conditions, again, there was no significant difference between the obese and control groups upon the addition of cumulative concentrations of substance P (Figure 3b) with an EC_50 _of 6.27 nmol/L (1.83 – 21.39) and 6.39 nmol/L (2.81 – 14.56) respectively.

**Figure 4:**
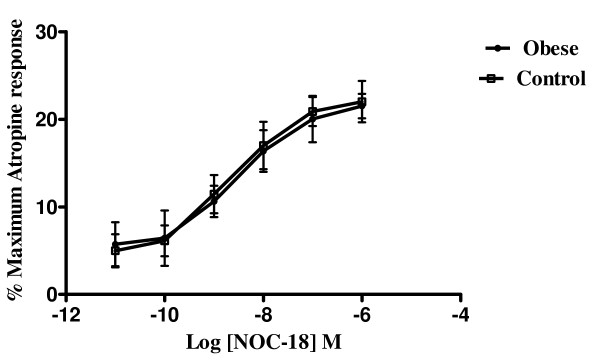
**Logarithm concentration-response curves of a nitric oxide donor (deta-nonoate) from obese and control patients**. Results shown are a mean of 6–8 patients in each group, expressed as a percentage of the maximum response to atropine (I micromol/L). A virtually identical graph was obtained when the experiment was performed under NANC conditions. There are no statistically significant differences demonstrated in either group.

Nitric oxide produced a concentration-dependant relaxation as expected. For these strips, the evoked contractions in from both control and obese patients were virtually abolished by the addition of 1 μmol/L atropine at the end of each experiment. This was then taken as 100% for each strip and all other calculations relating to that strip were recorded as a percentage of this value. In both groups (Figure 4), the release of nitric oxide lead to a concentration dependant relaxation of the tissues but there was no significant differences between the obese and control groups with either equivocal or NANC conditions (not shown), with an IC_50 _of 2.26 nmol/L and 2.05 nmol/L in the control and obese groups respectively under equivocal conditions and similar results when performed under NANC conditions (2.45 nmol/L and 2.13 nmol/L respectively).

## Discussion

A fundamental difference in contractility between obese and non-obese small bowel was found here. The initial differences seen in response to the muscarinic agonist carbachol, a parasympathomimetic that directly stimulates cholinergic receptors, demonstrate a significant difference in cholinergic responsiveness between the two groups. Because of the complexity of the multiple pathways involved in agonist-induced contraction including cross talk between cyclic nucleotides and their respective protein kinases, three very different agonists and also an inhibitor were chosen to evaluate the hypothesis. Prostaglandin F2a (a smooth muscle mitogen that promotes changes in smooth muscle contractility [7]), substance P (a tachykinin, the response to which is possibly regulated by the extra-cellular matrix of smooth muscle [8], and which may also act as a mediator of neurogenic inflammation in the gut [9].) and carbachol (a simple muscarinic agonist) act to cause smooth muscle contractility. This is via diverse mechanisms and yet, there are significant differences between obese and non-obese contractility for all three agonists. The fact that these differences are abolished under non-adrenergic, non-cholinergic conditions suggests that the differences are neurally mediated.

As expected, nitric oxide produced an inhibitory response [10], and the finding of no significant difference between obese and non-obese small bowel either under equivocal or NANC conditions adds further weight to the hypothesis that these differences are neurally mediated, as NO is known to act independently of adrenergic and cholinergic transmission [11].

This approach has the inherent limitations of any in vitro system. The absence in vitro of any blood supply to the tissue may have a considerable influence on the release, interaction with and removal of other transmitters not studied here. The pharmacological induction of contractility in the initial stages is artificial but by using each strip as its own control and for comparison, the inherent problems with standard electrical field stimulation to all strips may be lessened. Therefore, extrapolation to the clinical setting should be made with caution.

Whether altered intestinal contractility in the obese affects neurohormonal mechanisms of the control of satiety warrants further investigation. The alterations in the cholinergic responsiveness of intestinal smooth muscle in obesity as demonstrated in this study may result in altered intestinal motility, which in the clinical realm may suggest faster enteric emptying and more rapid intestinal transit. This in turn may translate into reduced satiety and enhanced appetite in the obese. This highlights intestinal pacing and pharmacological manipulation of small bowel contractility as potential therapeutic targets in the management of obesity in the future.

In summary, this study demonstrates that in vitro contractility in small bowel smooth muscle from obese patients, induced by common agonists, is significantly enhanced compared to that from non-obese patients. It is likely that these differences are adaptive in nature, but this again remains to be determined. Whether these differences are indeed neuronal in nature and whether they are central or peripheral in origin warrants further investigation. One possible key component to this may be the recently elucidated endocannabinoid system which is neurally-mediated and which is known to be over-active in obesity [12]

## Competing interests

The authors declare that they have no competing interests.

## Authors' contributions

TKG carried out all of the experimental work in this paper, prepared the statistical analysis and wrote the paper. AWB made significant contributions to the overall experimental design and to the conclusions and discussion in the paper. DCW made an invaluable contribution to the initial hypothesis and experimental design as well as to the overall analysis and interpretation of results.
